# Sphingolipidomics of serum in extremely preterm infants: Association between low sphingosine-1-phosphate levels and severe retinopathy of prematurity

**DOI:** 10.1016/j.bbalip.2021.158939

**Published:** 2021-04-20

**Authors:** Anders K. Nilsson, Mats X. Andersson, Ulrika Sjöbom, Gunnel Hellgren, Pia Lundgren, Aldina Pivodic, Lois E.H. Smith, Ann Hellström

**Affiliations:** a Department of Clinical Neuroscience, Institute of Neuroscience and Physiology, Sahlgrenska Academy, University of Gothenburg, Gothenburg, Sweden; b Department of Biology and Environmental Sciences, The Faculty of Science, University of Gothenburg, Gothenburg, Sweden; c Institute of Health and Care Sciences, Sahlgrenska Academy, University of Gothenburg, Gothenburg, Sweden; d Institute of Biomedicine, Sahlgrenska Academy, University of Gothenburg, Gothenburg, Sweden; e School of Medical Sciences, Faculty of Medicine and Health, Örebro University, Örebro, Sweden; f The Department of Ophthalmology, Boston Children’s Hospital, Harvard Medical School, Boston, MA, USA

**Keywords:** Ceramide, Hexosylceramide, Lipidomics, Phosphatidylcholine, Preterm birth, Sphingolipids

## Abstract

**Background:**

Extremely preterm infants are at risk of developing retinopathy of prematurity (ROP) that can cause impaired vision or blindness. Changes in blood lipids have been associated with ROP. This study aimed to monitor longitudinal changes in the serum sphingolipidome of extremely preterm infants and investigate the relationship to development of severe ROP.

**Methods:**

This is a prospective study that included 47 infants born <28 gestational weeks. Serum samples were collected from cord blood and at postnatal days 1, 7, 14, and 28, and at postmenstrual weeks (PMW) 32, 36, and 40. Serum sphingolipids and phosphatidylcholines were extracted and analyzed by LC-MS/MS. Associations between sphingolipid species and ROP were assessed using mixed models for repeated measures.

**Results:**

The serum concentration of all investigated lipid classes, including ceramide, mono- di- and trihexosylceramide, sphingomyelin, and phosphatidylcholine displayed distinct temporal patterns between birth and PMW40. There were also substantial changes in the lipid species composition within each class. Among the analyzed sphingolipid species, sphingosine-1-phosphate showed the strongest association with severe ROP, and this association was independent of gestational age at birth and weight standard deviation score change.

**Conclusions:**

The serum phospho- and sphingolipidome undergoes significant remodeling during the first weeks of the preterm infant’s life. Low postnatal levels of the signaling lipid sphingosine-1-phosphate are associated with the development of severe ROP.

## 1. Introduction

Infants born extremely preterm (less than 28 weeks of gestation) often develop morbidities that lead to lifelong physical and cognitive disabilities [1,2]. Advances in neonatal medicine over recent decades have significantly improved the survival of infants born at the lowest gestational ages (GA). However, higher rates of survival have led to an increase in morbidities that relate to the duration of the pregnancy [3,4]. Events taking place in the infant’s first days and weeks of life are crucial for later outcomes [5]. Understanding the metabolic processes and their regulation in infants born prematurely in this window of susceptibility are therefore of great clinical importance.

Retinopathy of prematurity (ROP) is a neurovascular eye disease that affects infants born preterm, particularly those born at low GA, and is a leading cause of childhood blindness globally [6]. In addition to low GA at birth, high and fluctuating oxygen supply, low birth weight, and poor postnatal weight gain have been identified as risk factors for severe ROP [7]. ROP progresses in two distinct phases. The first phase of ROP is induced by hyperoxia and low levels of growth factors such as insulin-like growth factor (IGF) and vascular endothelial growth factor (VEGF). This phase is characterized by an arrest of retinal vascularization. In the second phase of ROP, the avascular retina becomes hypoxic leading to stimulation of growth factor production and pathological retinal neovascularization. The degree of inhibition of normal vascular development determines the degree of release of hypoxia-induced factors that stimulate neovascularization. Therefore, improving normal retinal vascularization early in the development of ROP prevents later destructive neovascularization. Current treatments for ROP are designed to limit the neovascularization of the retina during the later phase of the disease and include laser photocoagulation and anti-VEGF injections in the eye. Both of these treatments have potentially adverse effects. Bio-markers for early detection of ROP and targets for preventative therapeutic interventions in early ROP development to limit disease progression are thus urgently needed.

**Fig. 1.**
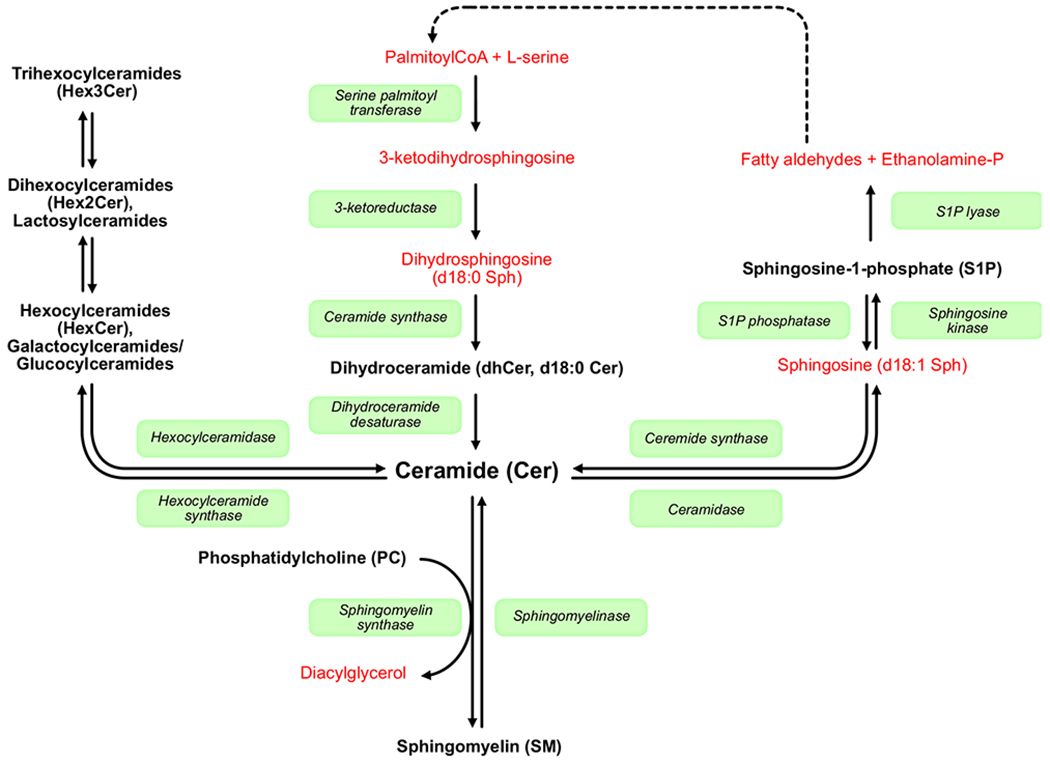
Simplified scheme of sphingolipid biosynthesis. Selected enzymes participating in the sphingolipid metabolism are shown in boxes, and lipids analyzed in this study are indicated in black bold letters. *De novo* sphingolipid synthesis is initiated by the condensation of fatty acyl-CoA and l-serine. Palmitoyl-CoA (16:0) is the favored substrate for this reaction, but other fatty acyl-CoAs, primarily myristoyl-CoA (14:0) and stearoyl-CoA (18:0), may also be used, as well as other amino acids. From the point of ceramide, the synthesis pathways branch out creating a wealth of complex sphingolipids, differing in their lipid backbones and head groups. The only exit for sphingolipids from this tree is by the activity of S1P lyase on sphingosine-1-phosphate, producing ethanolamine phosphate and fatty aldehydes. The fatty aldehydes can be recycled through a series of enzymatic steps (not shown) to reenter the *de novo* pathway or be used in other metabolic processes.

Lipids play many roles during fetal and neonatal development and alterations in lipid composition are associated with several preterm morbidities [8–11]. Sphingolipids constitute a structurally diverse class of bioactive lipids that include sphingomyelins (SM), ceramides (Cer), glycosphingolipids, and sphingosine-1-phosphate (S1P) (Fig. 1). Common to all sphingolipids is the presence of a long-chain sphingoid base that forms a lipid backbone. Sphingosine (d18:1) is the dominating long-chain base in sphingolipids found in human plasma, but species containing sphinganine (d18:0) and sphingadienine (d18:2) are also present [12]. Neonates can acquire sphingolipids from their enteral nutrition or synthesize them *de novo* from palmitoyl-CoA and serine. It is of great interest that about half of the polar lipids found in breast milk are sphingolipids; this proportion is however significantly lower in infant formulas as well as in parenteral lipid emulsions [13]. Studies on animal models have implicated a role for sphingolipids, and in particular, S1P in ROP. In mice, S1P modulates angiogenesis and vascular maturity [14]. Activation of S1P receptor 1 suppresses VEGF-dependent angiogenic sprouting, a key factor in the pathogenesis of ROP. In a mouse model of ROP (oxygen-induced retinopathy, OIR), S1P was recently shown to perhaps influence retinal vascularization [15]. Circulating S1P is derived from multiple sources including red blood cells (RBCs), platelets, and vascular endothelium [16]. While RBCs are the major contributor to S1P plasma levels, a considerable fraction of the S1P found in serum is derived from activated platelets and is released in response to coagulation after blood sampling [17]. S1P synthesis is stimulated by the omega-3 long-chain polyunsaturated fatty acid docosahexaenoic acid (DHA) [18], which in turn has been shown to protect against ROP [19,20]. Phosphatidylcholine (PC) is the main carrier of DHA in the blood of preterm infants [21]. To the best of our knowledge, no previous studies in preterm infants have corroborated the data obtained from animal models with a role for sphingolipids in neonatal ROP development.

Here, we studied early postnatal changes in the phospho- and sphingolipidome in the sera of extremely preterm infants and analyzed the relationship between these signaling lipids and the development of severe ROP, i.e. neovascularization and/or severe ROP needing treatment (Type 1 ROP).

## 2. Methods

### 2.1. Study design

This is a prospective study performed within the Donna Mega trial (NCT02760472), a monocentric randomized intervention study that compared two parenteral lipid emulsions, Smoflipid (Fresenius Kabi AB, Uppsala, Sweden) and Clinoleic (Baxter Medical AB, Kista, Sweden), with the primary outcome being ROP and its relation to infant serum fatty acid levels. Infants born at GA below 28 weeks admitted to the neonatal intensive care unit at Sahlgrenska University Hospital in Gothenburg between 201304-04 and 2015-09-22 were eligible for inclusion. Exclusion criterion was any major congenital malformation. In total, 78 infants were included in the original study. Out of these, 47 infants, where sufficient serum volumes for sphingolipid analysis remained after the Donna Mega trial’s primary analyses, were included in the present study. Details of the cohort and results of primary outcomes have been previously published [10,22].

### 2.2. Nutritional management

Details of the nutritional strategy have been previously published [22]. Briefly, parenteral nutrition with amino acids (Vaminolac, Fresenius Kabi, Uppsala, Sweden) and 10% glucose was initiated soon after birth, targeting a volume of 80–90 mL/kg/d during the first 24 h. Unless contraindicated, parenteral lipids (Clinoleic or Smoflipid) were introduced at 6 to 12 h after birth at a rate of 1 g/kg/d with daily increases up to 2 g/kg/d. Minimal enteral feedings with 1–2 mL of human milk per feeding were initiated within 3 h after birth, and volumes were gradually increased (10–20 mL/kg/d) until the goal volume of 160–180 mL/kg/d was reached. The first-hand choice for enteral feeds was expressed maternal milk, and if unavailable pasteurized donor milk was used. After postmenstrual age 34 weeks, preterm formula substituted the donor milk for infants where maternal milk was not available. When an enteral intake of 70 ml/kg/d had been reached, if required, the milk (maternal or donor) was fortified with bovine human milk fortifier (Nutriprem, Nutricia, France).

### 2.3. Ethics approval and consent to participate

The study was approved by the Regional Ethical Board at the University of Gothenburg, Göteborg, Sweden (Dnr. 303-11). Informed written consent for all participants was obtained from their parents or legal guardians.

### 2.4. ROP screening and classification

ROP examinations were performed according to a routine protocol that consisted of dilated ocular fundus examinations once or twice weekly. ROP was classified with the International Classification of Retinopathy of Prematurity [23]. No/moderate ROP was defined as ROP stages 1–2, and Severe ROP as ROP stage 3 and/or Type 1 ROP. Treatment was provided according to recommendations of the Early Treatment for Retinopathy of Prematurity Cooperative Group [24].

### 2.5. Blood sampling and cell count

For lipid analysis, blood was collected from the umbilical cord at birth and from veins postnatal days (PND) 1, 7, 14, and 28, and postmenstrual weeks (PMW) 32, 36, and 40. Samples were centrifuged within 2 h after collection, serum aliquoted, and stored at −80 °C. In total, 272 samples were analyzed with the following sample distribution across time points: Cord blood (CB) *n* = 21, PND 7 *n* = 39, PND 7 *n* = 36, PND 14 *n* = 33, PND 28 *n* = 32, PMW 32 *n* = 35, PMW 36 n = 35, PMW 40 *n* = 41. Multiple samples from the same individual were extracted together but infants with different ROP outcomes were mixed between lipid extraction batches to minimize batch effects.

Blood samples for platelet counts and hemoglobin (Hb) analysis were collected according to clinical indication. Mean daily platelet counts and Hb were calculated and used for correlation analyses with S1P in serum samples collected on the same day.

### 2.6. Lipid extraction

Lipids were extracted from 5 to 10 μL serum using butanol-methanol liquid-liquid extraction [25]. Serum was thawed on ice and added to 300 μL ice-cold n-butanol:methanol (3:1) containing butylated hydroxytoluene (BHT, 0.05% *w/v*) and internal standards d7S1P (10 pmol), Cer d18:1/17:0 (20 pmol), SM d18:1/12:0 (250 pmol), GalCer d18:1/12:0 (20 pmol), and PC 17:0/17:0 (200 pmol), in a 12 × 75 mm glass tube. All sphingolipid standards were from Avanti Polar Lipids (Alabaster, AL, U.S.) and the PC standard from Larodan AB (Solna, Sweden). Samples were sonicated in an ultrasonic bath for 30 min at 4 °C before phase separation was induced by the addition of 300 μL heptane:ethyl acetate (3:1) and 300 μL 1% acetic acid. After vigorous mixing, samples were centrifuged 10 min at 1500 *g* and the upper organic phase transferred to a new tube. The aqueous phase was re-extracted twice: the first time with 500 μl heptane:ethyl acetate (3:1) plus 50 μl n-butanol, and the second time with only 500 μl heptane:ethyl acetate (3:1). The pooled extracts were evaporated to dryness under N_2_ and dissolved in 50 μL methanol. For analysis of S1P and ceramides, a 25 μL sample was transferred to a 300 μL glass LC vial and 5 μL freshly prepared sodium methoxide was added to remove glycerolipid esters that could potentially cause ion suppression and interfere with quantification. The reaction was stopped by the addition of 1 μL concentrated formic acid.

### 2.7. LC-MS analysis

Lipids were analyzed on an Agilent 1260 Infinity HPLC system coupled to an Agilent 6470 triple quadrupole mass spectrometer (Agilent Technologies, Santa Clara, CA) equipped with a Jet Stream electrospray ionization source. PC and SM lipid species were separated on an Acclaim C30 2.1 × 50 mm 3-μm analytical column connected to a 2.1 × 10 mm 5-μm guard cartridge (ThermoFisher Scientific, Thermo Electron Sweden AB, Hägersten, Sweden) thermostated to 50 °C. Mobile phase A consisted of acetonitrile:methanol:water (35:35:30 by volume) and B isopropanol. Mobile phases were supplemented with 0.1% formic acid, 0.05% ammonia, and 5 μM phosphoric acid, and flows and gradient were as previously described. [26]. A 2 μL sample was injected on the LC system for the analysis. The electrospray ion source was operated at a temperature of 250 °C using N_2_ gas at a flow of 6 L min^−1^ and nebulizer pressure 20 psi. Sheath gas (N_2_) temperature was 200 °C and sheath gas flow 9 L min^−1^. All lipids were analyzed in positive mode with capillary and nozzle voltage set to 5000 V and 500 V, respectively. SM and PC species were detected using the MRM transitions previously described [26]. Principal fatty acyls in PC species were determined by analyzing an independent set of samples derived from adult serum using product ion mode in negative mode, where precursor ions were detected as their formic acid adducts [M + OAc]^−^ and fatty acids as [M]^−^.

S1P, ceramides, and hexosylceramides were separated on an InfinityLab Poroshell 120 EC-C18, 2.1 × 100 mm, 2.7-μm analytical column connected to a same-type 5 mm guard cartridge (ThermoFisher Scientific, Thermo Electron Sweden AB, Hägersten, Sweden) thermostated to 45 °C. Mobile phase A consisted of water and B methanol:isopropanol (4:1 by volume), both supplemented with 0.2% formic acid and 1 mM ammonium fluoride. Lipids were eluted using the following gradient: 75% mobile phase B for 2 min, linear increase to 100% B in 13 min and then held for 15 min, back to 75% B in 1 min, and finally re-equilibration for 6 min (total runtime 37 min) at a constant flow of 0.25 mL min^−1^. Samples were maintained at 10 °C in the LC system autosampler before analysis and the needle was washed with ACN:MeOH:IPA:H_2_O (40:40:15:5, by vol.) between injections. 5 μL sample was injected for the analysis of S1P and ceramides. Source gas temperature was 260 °C at a flow of 6 L min^−1^ and nebulizer pressure 35 psi. Sheath gas (N_2_) temperature was 200 °C and sheath gas flow 9 L min^−1^. The acquisition was in positive mode with capillary and nozzle voltage set to 4000 V and 500 V, respectively. MRM transitions, fragmentor, and collision energy settings for all analyzed lipids are listed in Table S1. Response curves were constructed using pure standards of S1P, Cer (d18:1/16:0, d18:1/20:0 and d18:1/24:1), and GlcCer (d18:1/18:0 and d18:1/24:1), with R^2^ ≥ 0.99 for all lipid standards. The intra-assay coefficient of variation (CV) for the method was for dhCer 9.2%, d18:1 Cer 11.5%, d18:2 Cer 8.0%, d18:0 HexCer 9.4%, d18:1 HexCer 10.2%, d18:2 HexCer 8.5%, d18:1 Hex2Cer 4.8%, d18:1 Hex3Cer 7.5%, SM 7.3%, and PC 6.3%. CV for S1P was 6.4%.

### 2.8. Statistical analyses

Student’s *t*-test was used for group comparison of data with normal distribution and Mann–Whitney *U* test for skewed data. The longitudinal values of 89 different sphingolipids were first analyzed in separate unadjusted analyses to identify those that were overall significantly associated with ROP category after adjustment for false discovery rate using the Benjamini-Hochberg procedure [27]. Each sphingolipid species was analyzed using mixed models for repeated measures data with lognormal distribution. Time (age in days) was modeled with the spline function with fixed knots in 0.1, 0.5, and 0.9 fractions of time. Within infant correlation was adjusted for in the random statement, using linear exponent autoregressive covariance structure. Each respective sphingolipid species was the dependent variable, No/moderate ROP vs Severe ROP, time (modeled with spline function), and interaction between the two were fixed effects with no further covariate adjustment in this first evaluation of all the 89 sphingolipid species. Diagnostic plots were reviewed and found satisfactory. The strongest related sphingolipid species, S1P, was further analyzed using the same method adjusted for GA at birth and time-updated weight standard deviation score (SDS) change from birth. Interaction between GA and time (spline), change in weight SDS and time (spline), as well as sex and birth weight alone and with interaction with time, were investigated but not found significant; hence not included in the adjustment. Difference between the estimated median values of S1P for time points PND 0 to 125 were computed, adjusting the confidence intervals (CIs) and *p*-values for multiple testing using Scheffe-Holm method. Longitudinal curves with 95% CI were created for the two ROP categories.

The correlation coefficient between S1P vs platelet count, Hb, and PC 38:6 based on longitudinal data was estimated using mixed models for repeated measures. Variable type, time point, and interaction between type and time point were modeled as fixed effects, assuming variables being linked over time and following the multivariate normal distribution. All four variables were analyzed in logarithmic scale, shown to be required for fulfilment of the assumption criteria for the method. Un-structured covariance matrix was used, from which the correlation of interest was obtained. The bootstrapping method with 1000 samples was applied to compute the p-value using the z-test.

All tests were two-sided. No data imputation was made. Statistical analyses were performed using IBM SPSS Statistics version 26 (IBM Corp, Armonk NY, USA) and SAS software version 9.4 (SAS Institute Inc., Cary, NC, USA).

## 3. Results

### 3.1. Study cohort

The study population included 47 infants born at less than 28 weeks GA. The baseline characteristics of the cohort are reported in Table 1. Infants who developed severe ROP (stage 3 with neovascularization) and/or Type 1 ROP had lower birth weight and were born at lower GA than infants who developed no or moderate ROP (stages 0–2).

### 3.2. Serum lipids display postnatal concentration changes

**Fig. 2.**
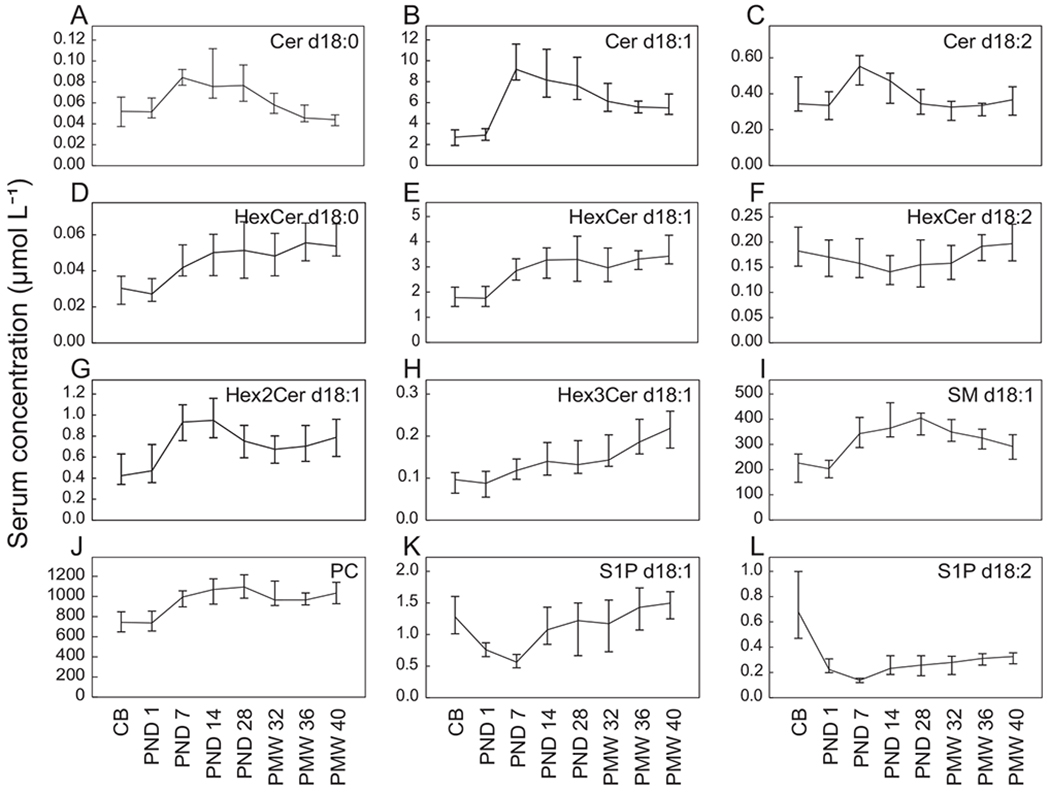
Sphingolipids and PC in infant serum. Plots show quantified lipid species summarized by lipid class over time point. CB, cord blood; Cer, ceramide; HexCer, hexosylceramide, Hex2Cer, dihexosylceramide; Hex3Cer, trihexosylceramide; PC, phosphatidylcholine; PND, postnatal day; PMW, postmenstrual week; S1P, sphingosine-1-phosphate; SM, sphingomyelin. Concentrations are reported as medians and 95% CI.

Serum samples collected at seven time points spanning from birth to term equivalent age were used for targeted LC-MS/MS lipidomics. Twenty-six PC species and 89 sphingolipid species covering 10 sphingolipid classes were quantified in all samples. Median serum concentrations for the whole study population by lipid class and time point are shown in Fig. 2. The concentration of sphingolipids (ceramides [Cer], mono-, di- and trihexocylceramides [HexCer, Hex2Cer and Hex3Cer], sphingomyelin [SM], and S1P) are reported according to their sphingoid base: sphinganine, sphingosine, and sphingadienine; d18:0, d18:1, and d18:2, respectively.

All analyzed lipid classes displayed clear longitudinal concentration changes. PC (Fig. 2J) and SM d18:1 (Fig. 2I) were, as expected, the two lipid classes present at the highest serum concentrations, followed by Cer d18:1 (Fig. 2B) and HexCer d18:1 (Fig. 2E). PC and SM levels increased from birth (CB) over the first month of life (PND 28) before decreasing until PMW 40.

Levels of all quantified Cer classes (Fig. 2A–C) displayed similar temporal patterns; concentrations increased from birth to PND 7 and then decreased until term equivalent age. In contrast, the analyzed HexCer classes demonstrated strikingly different temporal patterns. The postnatal changes in S1P species (d18:1 and d18:2, Fig. 2K and L) were distinctly different from the other lipids: concentrations decreased from that in cord blood to PND 7 before increasing throughout the study period.

### 3.3. Lipid species composition within lipid classes changes during early postnatal life

**Fig. 3.**
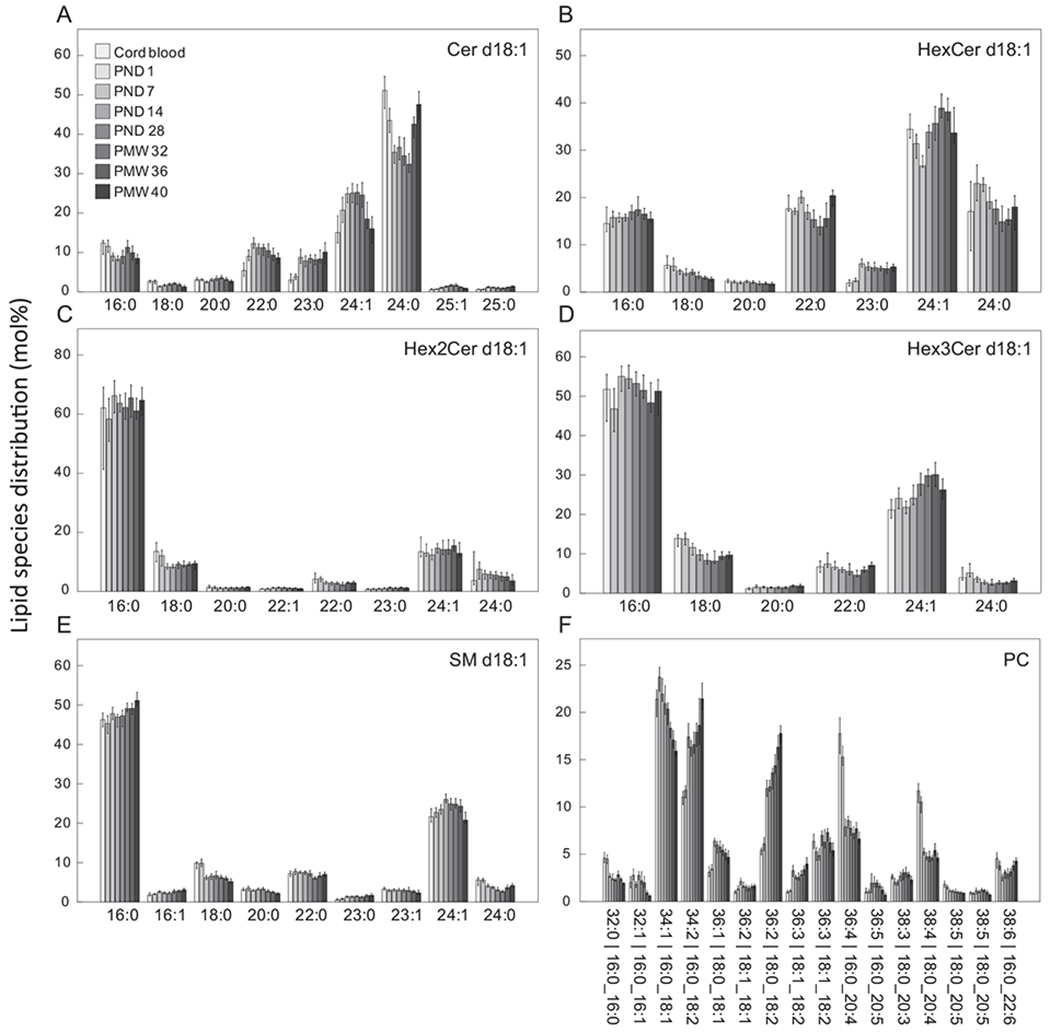
Postnatal changes in lipid species profiles. Shown are quantified lipid species >1 mol%. Bars represent medians and whiskers 95% CI. See Fig. S1 for additional lipid classes.

Next, we analyzed changes in the distribution of lipid species within each lipid class (Fig. 3 and S1) and interpreted patterns based on visual review. There were apparent changes in the species composition within all studied lipids classes across the study period. Many of the sphingolipid classes, including Cer (Fig. 3A), HexCer (Fig. 3B), and SM (Fig. 3E), displayed an inverse relationship in the proportion of species containing lignoceric acid (24:0, number of acyl carbons:number of acyl double bonds) and nervonic acid (24:1 n-9); when species containing lignoceric acid increased, the proportion of species with nervonic acid decreased, and vice versa. The proportion of lipid species carrying tricosylic acid (23:0) increased over time in all sphingolipid classes, while the proportion of species containing stearic acid (18:0) decreased.

Palmitic acid (16:0) was the primary acyl group in Hex2Cer (Fig. 3C), Hex3Cer (Fig. 3D), and SM (Fig. 3E) with sphingosine bases (d18:1), while lignoceric acid and nervonic acid were the dominating acyl group in all other analyzed sphingolipid classes (Fig. 3A, 3B and S1).

For PC (Fig. 3F), there was a time-dependent decrease in the proportion of 36:4 and 38:4 species, particularly apparent during the first postnatal week. These lipids species primarily correspond to acyls 16:0_20:4 and 18:0_20:4, i.e. palmitic or stearic acid in combination with arachidonic acid (20:4 n-6). The proportion of 38:6, containing palmitic acid and DHA (22:6 n-3), decreased during the first postnatal week and then gradually increased over time. Furthermore, there was a large increase over time in the proportion of PC 34:2 and one of the quantified 36:2 isomers, representing palmitic or stearic acid together with linoleic acid (18:2 n-6), respectively.

### 3.4. Lipid species associated with the development of severe ROP

The overall association of the 89 analyzed sphingolipid species to No/moderate ROP (stages 0–2) vs severe ROP (stage 3 and/or Type 1 ROP) was investigated using mixed models for repeated measures. As we have previously reported on the association between serum phospholipid-bound fatty acids and ROP in this cohort [10,22], we did not analyze PC species versus ROP here. After controlling for false discovery rate, four lipid species remained significantly associated with ROP category (Table 2 and Table S2). S1P (d18:1) was identified as the lipid species showing the strongest association with severe ROP. Due to this finding, and previous reports from animal models on a potential role for S1P in ROP pathogenesis, we focused further analyses on S1P.

### 3.5. Low serum S1P is associated with the development of severe ROP

**Fig. 4.**
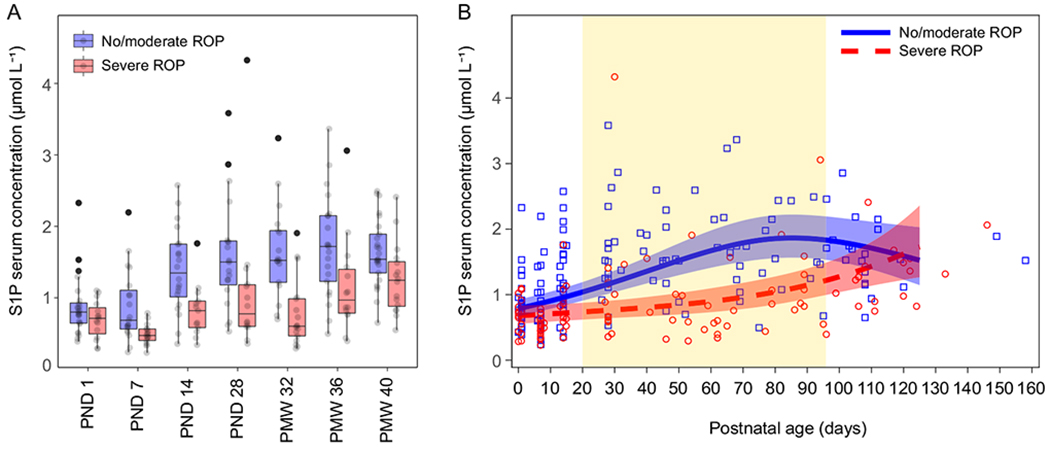
Levels of sphingosine-1-phosphate (S1P) in infant serum dichotomously grouped by final retinopathy of prematurity (ROP) stage, No/moderate ROP (stages 0–2, *n* = 28) and Severe ROP (stage 3 and/or Type 1 ROP, *n* = 19). A) Box plots with serum S1P level by time point. PND, postnatal day; PMW, postmenstrual week. B) Scatter plot of serum S1P levels by postnatal day of sampling and estimates from the mixed model for repeated measures adjusted for GA at birth and weight SDS. 95% CI are presented for the curves. Graph area highlighted in yellow represents time points where curves differ significantly after adjustment for multiplicity. The relative difference between Severe ROP and No/moderate ROP in the analysis adjusted for GA at birth and weight SDS change from birth over the study period (Scheffe-Holm adjusted *p*-values and 95% CIs) was: at 1 day, 0.86 (0.58–1.28) *p* = 0.77; at 14 days, 0.75 (0.53–1.07) *p* = 0.16; at 28 days, 0.66 (0.47–0.92) *p* = 0.0090; at 54 days, 0.54 (0.37–0.80) *p* = 0.0005; at 86 days, 0.58 (0.39–0.86) *p* = 0.0029; Overall test severe ROP vs No/moderate ROP *p* = 0.0002; Interaction TIME*GROUP *p* = 0.0013.

Fig. 4A shows the longitudinal serum concentration of S1P according to final ROP stages, no/moderate vs severe ROP at the pre-defined sampling time points. S1P concentration was significantly lower in infants who developed severe ROP at all time points except at PND 1 (PND 1, *p* = 0.194; PND 7, *p* = 0.002, PND 14, *p* = 0.006; PND 28, *p* = 0.014; PMW 32, *p* < 0.001; PMW 34, p = 0.014; PMW 40, *p* = 0.015).

Serum lipid composition is known to depend on GA at birth [28] and GA is an established confounder for the later development of ROP. Therefore, S1P association to ROP was tested with mixed models for repeated measures with postnatal days as time scale and adjusted for GA at birth and weight SDS change from birth over the study period (Fig. 4B). This demonstrated that S1P was significantly lower in infants who developed severe ROP, also after adjusting of GA and weight SDS change (overall test severe ROP vs no/moderate ROP, *p* = 0.0002).

Finally, there was no difference in S1P levels between infants who received Clinoleic and Smoflipid as parenteral lipid emulsion (data not shown).

### 3.6. Serum S1P is correlated with platelet count and serum PC 38:6 (PCDHA) but not Hb

**Fig. 5.**
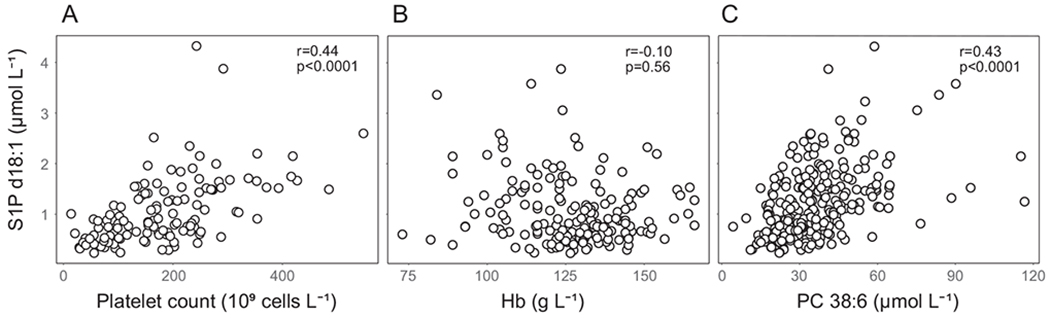
Correlations between S1P and A) platelet count, B) Hb, and C) PC 38:6 (16:0_22:6). Correlation coefficients were obtained from the mixed model for repeated measures procedure, linking each pair of the variables over time and assuming multivariate normal distribution. The variables were analyzed in logarithmic scale to fulfill the required assumptions for the method.

Infant serum S1P correlated well with mean daily platelet count but not with Hb (Fig. 5A and B). Furthermore, serum S1P levels showed a good correlation with PC 38:6, the main carrier of DHA in infant serum (Fig. 5C).

## 4. Discussion

The transition from intra- to extrauterine life is defined by large-scale metabolic changes; in part as a necessity for the neonate to cope with the new environment, and also a reflection of the interruption of the maternal-fetal transfer of nutrients [29]. In this study, we show that this transition is accompanied by a global remodeling of the serum sphingolipidome and that low neonatal level of the bioactive sphingolipid S1P is associated with abnormal retinal neurovascular development, i.e. severe ROP. This is to the best of our knowledge the first report following early longitudinal changes in the sphingolipidome of preterm infants.

Virtually all of the analyzed serum lipids displayed concentration changes during the time between birth and term equivalent age. Several aspects may contribute to these changes. After birth, lipids replace glucose as the primary energy source for the neonate. While maternal milk is the primary lipid source for a healthy term infant, all extremely preterm infants require the addition of parenteral lipids for some time before full enteral feeding is achieved. Therefore, external lipid sources together with endogenous metabolic processes contribute to the infants’ lipidome.

The importance of long-chain polyunsaturated fatty acids, particularly the n-3 fatty acid DHA and n-6 fatty acid arachidonic acid (AA), for preterm infant health and development is becoming increasingly recognized. It was recently reported that enteral supplementation with an oil enriched in DHA and AA to extremely preterm infants lowered the risk of developing severe ROP by more than 50% [20]. In this study, we observed a dramatic decrease in the fraction of PC species in serum containing AA and DHA during the first postnatal week. This is consistent with the previously published analysis of serum phospholipid-bound fatty acids by GC–MS in the same cohort [22]. Further, the proportion of PC containing linoleic acid increased throughout the study period. This also corroborates our previous findings on the phospholipid fatty acid profiles in these preterm infants [30] and reflects the high nutritional intake of linoleic acid during the neonatal period [31]. As expected, the PC species composition changes largely follow that of total phospholipid-bound fatty acids, as PC constitutes the major phospholipid class in serum. The serum total PC concentration, as well as changes in PC species composition during the first week of life in our study, were comparable to that reported in a similar cohort recently [32]. However, total PC concentration was somewhat lower, but SM concentration higher in this study than reported for a cohort of slightly less premature children [21]. Methodological differences and dissimilar nutritional strategies may contribute to this discrepancy. However, temporal profiles of PC and SM seem to be coherent between studies: an increase during the first month of life before a slight decrease towards the end of the study period. This is also consistent with observations on the postnatal concentration of serum lipoproteins in full-term infants [33]

We analyzed serum levels of sphingolipids with sphinganine (d18:0), sphingosine (d18:1), and sphingadienine (d18:2) sphingoid long-chain bases. Quantitatively, these long-chain bases represent ~80% of the total long-chain bases in human plasma [34]. During *de novo* synthesis, the double bond in sphingosine (Δ4E) is introduced by dihydroceramide desaturase 1 (DEGS1) whereas the additional double bond in sphingadienine (Δ14Z) is introduced by fatty acid desaturase 3 (FADS3) [34,35]. Dihydroceramides and ceramides have been shown to have non-overlapping biological effects in cell cycle progression, apoptosis, autophagy, and lipid homeostasis (reviewed in [36]) whereas the function of sphingadienine ceramides has been less studied. Interestingly, in baboon neonates, dietary DHA and AA upregulate the expression of FADS3 [37], possibly influencing the d18:1/d18:2 ceramide ratio. While the sphingoid backbone will affect the biophysiochemical properties of the lipid molecule, function is also determined by the chain length and the degree of unsaturation of the fatty acid linked to the sphingoid moiety.

Both the within-class composition of sphingolipids as well as the total concentration of the different sphingolipid classes displayed unique temporal profiles. As most infants received full enteral feeding with human milk after the first two weeks of life [30], many of the changes in serum lipid composition occurring beyond this point are likely the result of endogenous lipid metabolism. Beside palmitic acid (16:0), the dominating acyls in most sphingolipid classes were lignoceric (24:0) and nervonic acid (24:1 n-9). There appeared to be an inverse relationship in the proportion of species containing lignoceric acid and nervonic acid. For example, among the d18:1 ceramides and d18:1 SMs, the proportion of lignoceric acid increased while nervonic acid decreased between PMW 32 and term equivalent age (PMW 40). During the same period, both lignoceric acid and nervonic acid declined significantly in the maternal milk [38], suggesting that infant levels of these acyls do not directly reflect dietary intake.

There is little longitudinal data on sphingolipids for full-term infants with which to compare our data. In a study comparing lipid profiles in cord blood from late preterm and term infants, the abundance of 39% of the analyzed lipid species was found to depend on GA at birth [28]. However, in the study by McCloskey et al., none of the analyzed sphingolipid classes (Cer, gangliosides, HexCer, and SM) were associated with GA at birth after adjusting for birth weight, gender, and labor. Notably, on a lipid species level, Cer d18:1/24:1 and Cer d18:0/24:1, as well as several sphingolipid species containing lignoceric acid, displayed a GA dependence in cord blood. We did not analyze cord blood levels of sphingolipids in relation to GA due to the low number of samples (*n* = 21).

Sphingolipids are essential for fetal and infant brain development. In a small pilot study, the administration of SM-fortified milk to very low birth weight infants was positively associated with neurobehavioral development [39]. Furthermore, supplementing formula to full-term infants with bovine milk fat globule membranes (enriched in phospho- and sphingolipids) has been associated with positive neurological outcomes [40] and altered infant lipid profiles, particularly affecting the serum composition of PC, SM, and Cer lipid species [41]. It is not fully understood how circulating levels of sphingolipids reflect those found in peripheral organs. A study on mice points to that the relation between sphingolipids in plasma and brain tissue is complex, and both positive and negative correlations were found between ceramides in plasma and hippocampus/cerebral cortex [42]. It has been proposed that the level of nervonic acid in SM in RBCs of preterm infants reflects brain maturation and myelination [43]. Our data of serum sphingolipids do not support this assessment as the level of nervonic acid in SM is only transiently increased during the time from birth to PMW 40.

S1P was identified as the lipid species showing the greatest association with severe ROP. The association between low serum S1P and severe ROP remained significant also after adjusting for GA at birth and weight SDS change. S1P is a potent lipid mediator with well-described and versatile biological roles mediated through its binding with S1P receptors 1–5 (S1PR_1–5_). S1P exerts both pro and anti-angiogenic effects. Specifically, S1P and S1PRs are essential for retinal vessel development and emerging data indicate that S1P signaling has a prominent role in ROP pathogenesis. In the mouse OIR model for ROP, overexpressing the sphingosine kinase (Sphk2), and thereby upregulating retinal S1P, resulted in increased retinal vascularization under physiological conditions and chaotic neovascularization in OIR [15]. In mice lacking Sphk2, conversely, retinal angiogenesis and neovascularization were reduced. In the same OIR model, mice lacking S1PR_2_ showed reduced pathological retinal neovascularization and displayed normal vascular development, concomitant with a diminished inflammatory response [44]. Furthermore, pharmacological inhibition of SIP through intraocular injections suppressed retinal neovascularization in mouse OIR [45]. While studies on animal models have identified S1P as an important mediator during or after the induction of pathological neo-vascularization, we found that serum S1P levels were lower in infants who developed severe ROP several weeks before the disease was diagnosed. S1P that is needed for normal vascularization might be protective for later retinal hypoxia that increases levels of growth factors such as VEGF, contributing to pathological neovascularization.

Adults exposed to high altitude hypoxia increase their Sphk1 activity in RBCs leading to elevated S1P, a mechanism that functions independently of S1PRs [46]. In turn, intracellular S1P promotes RBC glycolysis and O_2_ release, thereby ameliorating tissue hypoxia [46]. High serum S1P observed in our study in healthy infants may contribute to proper oxygenation of peripheral tissues in the early postnatal life, thereby reducing the later risk of ROP development.

Supplementation of DHA to adults was recently reported to increase levels of plasma S1P [47], and DHA is also known to enhance the expression of Sphk in vitro [18]. In line with this, serum PC 38:6, which is the main carrier of DHA in preterm infants, was positively correlated with S1P. Thus, the reported benefits of high blood levels of DHA for protection against ROP in preterm infants may be, at least in part, mediated through the action of S1P.

Activation of platelets, e.g. during blood clotting, leads to the release of their intracellular stores of S1P [17]. Therefore, the S1P concentration in serum is higher than that in plasma. We observed a positive correlation between the mean platelet count and serum S1P concentration in our samples but found no correlation between serum S1P and Hb, which is linearly related to RBC count. These results suggest that S1P levels partly reflect the number of platelets in circulation. We previously reported that low platelet counts and serum DHA in infants during the neovascularization phase in ROP were associated with the development of severe ROP [48,49]. S1P derived from platelets may in part explain why high platelet count is associated with favorable ROP prognosis.

In conclusion, we show that the sphingolipidome of extremely pre-term infants is dynamic and undergoes dramatic compositional changes during the time between birth and term equivalent age. Low serum levels of S1P were associated with abnormal retinal neurovascular development. Further studies are needed to elucidate the causality between S1P and other lipidomic changes on one hand, and the development of severe ROP on the other hand.

## Supplementary Material

supplementary tables

2

## Figures and Tables

**Table 1:** Demographic characteristics of the cohort.

Variable	All	No/moderate ROP	Severe ROP	p (no/moderate vs severe ROP)
Infants, n	47	28	19	
Samples, n	272	159	113	
Boys, n (%)	28 (60)	15 (54)	13 (68)	0.309
BW, g [mean (SD)]	762 (222)	862 (217)	617 (133)	<0.001
BW SDS [mean (SD)]	−1.13 (1.45)	−0.96 (1.52)	−1.38 (1.34)	0.325
GA at birth, wks [mean (SD)]	25.0 (16)	25.7 (1.4)	24.0 (12)	<0.001
Receiving Smoflipid, n (%)	27 (57)	17 (61)	10 (53)	0.582

BW=birth weight; ROP = retinopathy of prematurity; no/moderate ROP = no ROP or ROP stages 1 or 2; severe ROP = ROP stage 3 and/or type 1 ROP; SD = standard deviation; SDS = standard deviation score.

**Table 2:** Associations between longitudinal concentrations of lipids species in serum and severe ROP.

Lipid species	Overall p-value from MMRM	FDR adjusted p-value
S1P d18:1	<0.0001	0.0001679
S1P d18:2	<0.0001	0.0020116
Hex3Cer d18:1/18:0	0.0001	0.0041025
Cer d18:1/20:1	0.0020	0.044326

MMRM = mixed models for repeated measures; FDR = false discovery rate.

## Abbreviations:

PC: phosphatidylcholine
SM: sphingomyelin
Cer: ceramide
HexCer: hexosylceramide
Hex2Cer: dihexosylceramide
Hex3Cer: trihexosylceramide
S1P: sphingosine-1-phosphate
