# Supplementary material for: Sphingolipidomics of serum in extremely preterm infants: Association between low sphingosine-1-phosphate levels and severe retinopathy of prematurity
Source: Biochim Biophys Acta Mol Cell Biol Lipids. Author manuscript; Available in PMC 2021 Dec 1. (PMC8633973; doi:10.1016/j.bbalip.2021.158939)
Supplement: 2 [file NIHMS1756979-supplement-2.pdf]

Sphingolipidomics of serum in extremely preterm infants: association between low sphingosine-1-phosphate levels and severe retinopathy of prematurity

Anders K Nilsson[1], Mats X Andersson[2], Ulrika Sjöbom[1,3], Gunnel Hellgren[1,4], Pia Lundgren[1,5], Aldina Pivodic[1], Lois E Smith[6], and Ann Hellström[1]

1. Department of Clinical Neuroscience, Institute of Neuroscience and Physiology, Sahlgrenska Academy, University of Gothenburg, Gothenburg, Sweden
2. Department of Biology and Environmental Sciences, The Faculty of Science, University of Gothenburg, Gothenburg
3. Institute of Health and Care Sciences, Sahlgrenska Academy, University of Gothenburg, Gothenburg, Sweden
4. Institute of Biomedicine, Sahlgrenska Academy, University of Gothenburg, Gothenburg, Sweden
5. School of Medical Sciences, Faculty of Medicine and Health, Örebro University, Örebro, Sweden
6. The Department of Ophthalmology, Boston Children's Hospital, Harvard Medical School, Boston, MA, USA

Address correspondence to Anders K. Nilsson, anders.k.nilsson@gu.se

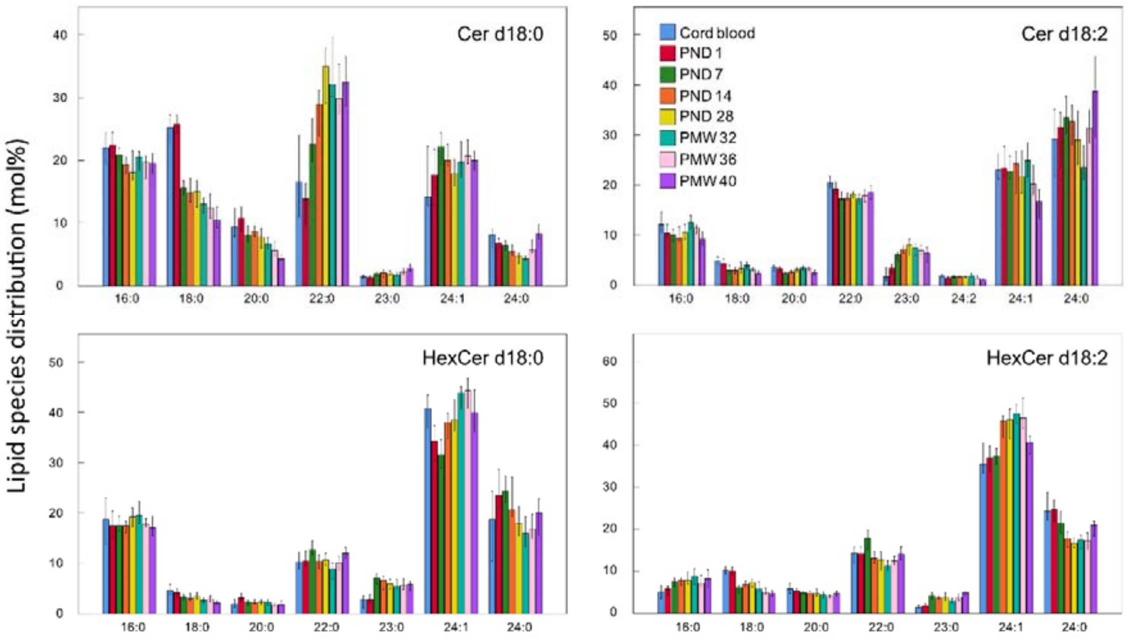

Figure S1. Postnatal changes in lipid species profiles. Shown are quantified lipid species >1 mol%. Bars represent medians and whiskers 95% CI.
